# Theranostic nanosensitizers for highly efficient MR/fluorescence imaging‐guided sonodynamic therapy of gliomas

**DOI:** 10.1111/jcmm.13811

**Published:** 2018-08-29

**Authors:** Hongmei Liu, Meijun Zhou, Zonghai Sheng, Yu Chen, Chih‐Kuang Yeh, Wenting Chen, Jia Liu, Xin Liu, Fei Yan, Hairong Zheng

**Affiliations:** ^1^ Department of Ultrasonography Guangdong Second Provincial General Hospital Guangzhou China; ^2^ Paul C. Lauterbur Research Center for Biomedical Imaging Institute of Biomedical and Health Engineering Shenzhen Institutes of Advanced Technology Chinese Academy of Sciences Shenzhen China; ^3^ State Key Laboratory of High Performance Ceramic and Superfine Microstructures Shanghai Institute of Ceramics Chinese Academy of Sciences Shanghai China; ^4^ Department of Biomedical Engineering and Environmental Sciences National Tsing Hua University Taiwan; ^5^ School of Life Science and Engineering Southwest University of Science and Technology Mianyang China; ^6^ Department of Ultrasonography Guangdong Provincial Hospital of Chinese Medicine The Second Clinical College of Guangzhou University of Chinese Medicine Guangzhou China

**Keywords:** glioma, imaging‐guided therapy, sinoporphyrin sodium, sonodynamic therapy, theranosticagents

## Abstract

Glioma is the most frequent primary brain tumour of the central nervous system. Its high aggressiveness and deep‐seated brain lesion make it a great challenge to develop a non‐invasive, precise and effective treatment approach. Here, we report a multifunctional theranostic agent that can integrate imaging and therapy into a single nano‐platform for imaging‐guided sonodynamic therapy (SDT). The SDT agents were fabricated by encapsulation of sinoporphyrin sodium (DVDMS) chelating with manganese ions into nanoliposomes (DVDMS‐Mn‐LPs). DVDMS‐Mn‐LPs are physiologically stable and biologically compatible, and they can produce singlet oxygen upon ultrasound irradiation to kill cancer cells. Both cell and animal studies demonstrated that SDT with DVDMS‐Mn‐LPs can significantly improve the antitumour growth efficiency even in the presence of skull. In addition, DVDMS‐Mn‐LPs are good for MR and fluorescence imaging. Thus, DVDMS‐Mn‐LPs reported here may provide a promising strategy for imaging‐guided modality for glioma treatment.

## 1 INTRODUCTION

Glioma is the most common primary malignant brain tumour and considered one of the most difficult curable tumours because of its typical characteristics such as rapid proliferation, strong invasiveness and unsatisfactory prognosis.^1^, ^2^, ^3^ The median survival for patients is only 12‐18 months.^4^ In clinic practice, surgery is traditionally the initial definitive treatment option for glioma patients. Although radiotherapy, chemotherapy or their combination treatments are also widely used for consolidating the surgical effect, they indeed cause a range of side effects and seriously lower the quality of the patient's life due to their non‐selective killing of malignant and normal cells.^5^, ^6^ Therefore, it is desirable to develop alternative or complementary glioma treatment modalities for safer and more efficient killing of tumour cells that may help to extend the patient survival.

To date, massive tumour treatment strategies have emerged, including photothermal therapy (PTT), photodynamic therapy (PDT), high intensity‐focused ultrasound (HIFU), radiofrequency ablation (RFA) and cryoablation.^7^, ^8^, ^9^ Most of them exhibit satisfactory efficacy against various types of tumours and actually some patients are benefiting from them, such as HIFU for uterine fibroids and RFA for liver cancer.^10^, ^11^ However, most of these modalities are not so effective for glioma treatment. An important reason may attribute to the violent histologic change from these treatments.^12^, ^13^ These changes are usually hazardous and disabling for patients because glioma is seated in the brain, with a huge number of nerve cells around the tumour. Clinical evidence has shown that heating of brain tissue in the sonication path resulted in a secondary focus outside the target causing neurological deficit.^12^

Photodynamic therapy is a mild treatment strategy, killing tumour cells through reactive oxygen species (ROS) generated from low‐intensity laser irradiation on photosensitizers.^14^ However, light cannot penetrate the skull, largely limiting its application in glioma treatment. Compared to PDT, sonodynamic therapy (SDT) is another approach for cancer treatment which uses ultrasound to activate sonosensitizers to generate ROS to kill cancer cells.^15^, ^16^, ^17^ Different from invasive RFA and cryoablation, ultrasound is featured with non‐invasiveness and high tissue penetrability, making it possible to reach and treat deep‐seated tumours, especially for glioma. More importantly, ultrasound can be focused into the brain in a small volume, achieving a precise position on tumours.^18^, ^19^, ^20^, ^21^ Although ultrasound can pass through the skull, it is of great challenge to precisely activate the sonosensizers accumulated in the glioma for efficient SDT. Imaging‐guided therapy has provided a new solution to solve this issue.^22^, ^23^, ^24^, ^25^, ^26^, ^27^ The imaging guidance plays unique roles and shows its intrinsic advantages in cancer treatment, especially for glioma. On the one hand, it could provide useful information such as size and location of the tumour, as well as the relationship of the tumour with surrounding tissues, which can help ultrasound for determining the irradiation position and scope. On the other hand, it could also provide the optimal time window for treatment so that the ultrasound irradiation could be conducted when the sonosensitizer reaches the peaked level in the targeted lesion. On this ground, we herein report a multifunctional theranostic agent that integrates imaging and therapy functionalities into a single nano‐platform for highly efficient imaging‐guided SDT. Organic sinoporphyrin sodium (DVDMS) molecules were initially used as the sonosensitizer since they have been widely used in PDT and SDT for the therapy of many human tumours.^28^, ^29^, ^30^ Paramagnetic Mn centres were further anchored into DVDMS molecules (DVDMS‐Mn) by directly chelating manganese ion (Mn^2+^) into DVDMS. To improve the delivery efficiency, the multifunctional sonosensitizer DVDMS‐Mn were further encapsulated into liposomes via a typical thin‐film rehydration method to fabricate DVDMS‐Mn‐Liposomes (DVDMS‐Mn‐LPs). The high performance of nanosized DVDMS‐Mn‐LPs for efficient imaging‐guided SDT against glioma has been systematically investigated and demonstrated both in vitro and in vivo.

## 2 MATERIALS AND METHODS

### 2.1 Materials

Sinoporphyrin sodium (DVDMS) was provided by Jiangxi Qinglong Group Co. Ltd (Jiangxi, China). Dipalmitoylphosphatidylcholine (DPPC) and 1, 2‐Distearoyl‐sn‐glycero‐phosphoethanolamine‐N‐[met‐hoxy (poly‐ethyleneglycol)‐2000] (DSPE‐PEG2000) were purchased from Avanti Polar Lipids Inc (Alabaster, AL, USA). Cholesterol, 2′,7′‐dichlorofluorescin diacetate (DCFH‐DA) and 4′,6‐diamidino‐2‐phenylindole (DAPI) were purchased from Sigma‐Aldrich (St. Louis, MO, USA). Fetal bovine serum (FBS), high glucose DMEM, trypsin‐EDTA and penicillin‐streptomycin were purchased from Gibco Life Technologies (Aargau, Switzerland). Cell Counting Kit‐8 (CCK‐8) was purchased from Dojindo Laboratories (Tokyo, Japan). Calcein‐AM and Propidium iodide (PI) were purchased from Invitrogen (Eugene, Oregon, USA). TdT‐mediated dUTP nick end labelling (TUNEL) kit was purchased from Roche Company (Shanghai, China). Anti‐PCNA antibody was purchased from Abcam (Cambridge, UK). Anti‐Ki‐67 antibody was purchased from Santa Cruz Biotechnology Inc (California, USA). The U87 human glioma cell line was purchased from the American Type Culture Collection. Female BALB/c athymic nude mice were obtained from Beijing Weitonglihua Experimental Animal Technology Co. Ltd (Beijing, China).

### 2.2 Synthesis of DVDMS‐Mn and preparation of DVDMS‐Mn–LPs

To chelate manganese with DVDMS, manganese chloride (MnCl_2_) was added to the DVDMS solution at a molar ratio of 2:1 (MnCl_2_:DVDMS) for 1 hour. Excess MnCl_2_ was removed using a dialysis process (MWCO = 1 kD). To prepare DVDMS‐Mn‐LPs, DPPC, Cholesterol and DSPE‐PEG_2000_ were dissolved in chloroform in a 55:40:5 molar ratios. The chloroform was removed under a nitrogen flow until uniform phospholipid films were formed. The phospholipid films were further dried for over 3 hour under vacuum, followed by hydration at 65°C with double‐distilled water containing DVDMS‐Mn solution (DVDMS = 1 mg/mL) to obtain a final total lipid concentration of 20 mg/mL. After hydration, the mixed solutions were sonicated in a bath ultrasonic oscillator until it became pellucid. To remove the unbound fraction of DVDMS‐Mn, the liposomes were ultracentrifuged at 100 000 *g* for 30 minutes at 4°C.

### 2.3 Characterization of DVDMS‐Mn‐LPs

The resulting DVDMS‐Mn‐LPs were subjected to TEM scanning (Tecnai G2 F20 S‐Twin; FEI, Hillsboro, OregonState, USA) and AFM scanning (MFP‐3D‐BIO; Oxford Instruments, Oxford, UK) to determine the particle morphology. The average diameter, zeta potential and size distribution of DVDMS‐Mn‐LPs were determined by dynamic light scattering (Zetasizer Nano Zeta; Malvern Instrument, Malvern, UK). The stability of DVDMS‐Mn‐LPs was assessed by determining the average diameter, zeta potential and PDI at predetermined times. In order to determine their photoproperties, DVDMS‐Mn‐LPs were subjected to the absorption spectra (Lambda 750; PerkinElmer, Waltham, Massachusetts, USA) and fluorescence spectrum (PE LS55; PerkinElmer) measurement. To determine encapsulation efficiencies (EE) of DVDMS, the free DVDMS in the supernatant was removed and measured by UV–vis spectrometer at *λ*_max_ of 359 nm. The encapsulation efficiencies (EE) of DVDMS were calculated according to the following formula: EE (%) = (DVDMS_total_ − DVDMS_free_) / DVDMS_total_ × 100%. The concentration of manganese in the DVDMS‐Mn‐LPs was measured by inductively coupled plasma atomic emission spectroscopy (ICP‐AES, OPTIMA 7000DV; PerkinElmer). To explore the possibility of using DVDMS‐Mn‐LPs as a T_1_‐weighted MR contrast agent, DVDMS‐Mn‐LP solutions with different Mn concentrations ranging from 0.008 to 0.32 mmol/L were scanned under a 3.0 T clinical MR scanner (TIM TRIO, Siemens, Germany) at room temperature. After acquiring the T_1_‐weighted MR images, the signal intensity of each sample was measured by drawing regions‐of‐interest. By plotting the *r*_1_ value (1/T_1_) for each sample against the Mn concentration, the final *r*_1_ value for DVDMS‐Mn‐LPs was calculated by curve fitting.

### 2.4 Detection of ROS in DVDMS‐Mn‐LPs

2′,7′‐dichlorofluorescin diacetate, which was highly sensitive to ROS, was employed here during the detection process. Samples containing DVDMS‐Mn‐LPs (DVDMS = 1 mg/mL) were mixed with 1 mmol/L DCFH‐DA, and then irradiated by different U.S. times or U.S. intensities. The level of ROS was determined by measuring enhanced fluorescence of dichlorofluorescein (DCF; *λ*_excitation_ = 494 nm and *λ*_emission_ = 521 nm) using a fluorescence spectrometer (FS920; Edinburgh Instruments, Edinburgh, UK).

### 2.5 Cellular uptake

The U87 human glioma cells were cultured in high glucose DMEM with 10% (v/v) foetal bovine serum, 1% (v/v) penicillin and 1% (v/v) streptomycin. Cells were incubated in a humidified incubator at 37°C with 5% CO_2_. For the cellular uptake experiment, U87 cells (2 × 10^5^ cells per well) were seeded in 6‐well plates and incubated overnight, and then incubated with DVDMS‐Mn‐LPs or free DVDMS (DVDMS = 1 μg/mL). After incubation for predetermined time, cells were rinsed with PBS three times, and fixed with 4% paraformaldehyde solution and incubated for 20 minutes each. The nuclear dye DAPI was used as a control to stain nuclei in the experiment. Images of cells were acquired using a biological inverted microscope (Olympus IX71, Olympus Corporation, Olympus, Japan). For quantitative analysis, these cells were harvested and determined by Accuir C6 flow cytometer using CFlow Plus software (BD, Ann Arbor, MI, USA).

### 2.6 Biocompatibility assay

U87 cells were cultured in standard cell media. Cells were first seeded into 96 well plates and then incubated with free DVDMS or DVDMS‐Mn‐LPs with different concentrations for 24 hour. The standard CCK8 assay was used to determine relative cell viabilities.

### 2.7 In vitro cell SDT

Six groups were involved in this study, including the untreated control group, liposome group (DVDMS‐Mn‐LPs), only ultrasound group (US), SDT group (DVDMS‐Mn‐LPs with ultrasound irradiation), SDT plus skull group (SDT + skull), and PDT plus skull group (PDT + skull). SDT + skull and PDT + skull groups mean that the skull was positioned in the beam pathway during SDT/PDT treatment (Figure S1). The ultrasound device composed of ultrasonic electronic generator (AFG3102; Tektronix, Beaverton, OregonState, USA), amplifier (AR150A100B RF/microwave instrumentation, E‐Tech industrial co., Shenzhen, China) and transducer (Valpey Fisher, Shenzhen, China), was shown in Figure S1A. The unfocused ultrasound transducer (diameter: 3.5 cm, resonance frequency: 0.5 MHz) was fixed at the bottom of water bath. The ultrasound irradiation was performed with the following parameters: pulse wave, 0.5 MHz transmit frequency, 50% duty cycle, 100 ms interval time, 10 Hz repetition and 0.5 W/cm^2^ ultrasound intensity for 5 minutes. The cell culture plate was suspended in the far field of ultrasound transducer (about 10 cm). The laser device was shown in Figure S1B. A 660 nm laser at a power density of 0.5 W/cm^2^ was used to irradiate these samples for 5 minutes.

The whole experiment was conducted in the dark to avoid phototoxicity. The SDT effects of DVDMS‐Mn‐LPs on U87 cells were verified using Calcein AM and propidium iodide (PI) co‐staining. U87 cells (2 × 10^5^ cells per well) were seeded in 6‐well plates and incubated overnight. Then the cells were incubated with DVDMS‐Mn‐LPs (DVDMS = 20 μg/mL) or PBS for 3 hour. Afterward, the cells of experimental groups were rinsed again with PBS and immersed in 2 mL fresh culture medium, and subsequently irradiated by ultrasound or laser with intensity of 0.5 W/cm^2^ for 5 minutes. After other 6 hour incubation, cells were stained with calcein‐AM for visualization of live cells and with PI for visualization of dead/late apoptotic cells. Afterward, these cells were rinsed again with PBS and were examined by an inverted microscope (Olympus IX71). For quantitative analysis, the cell viability was further verified by standard CCK8 assay.

### 2.8 In vitro cellular ROS detection

As for single oxygen detection, the probe reagent (DCFH‐DA) was dissolved in DMSO at the concentration of 10 mmol/L, and then diluted with PBS at the final concentration of 25 μmol/L for experiment use. U87 cells (1 × 10^4^ cells per well) were seeded in 96‐well plates and incubated overnight. Then the cells were incubated with DVDMS‐Mn‐LPs (DVDMS = 20 μg/mL) or PBS. After being incubated for 3 hour, the cells were washed and treated with the prepared probe solution for 1 hour. Subsequently, the probe solution was removed and the cells were treated as stated above. Single oxygen radicals were immediately measured using a multimode plate reader at the excitation wavelength of 490 nm and emission wavelength of 521 nm.

### 2.9 Animals and tumour model

Animals received care in accordance with the Guidance Suggestions for the Care and Use of Laboratory Animals. The procedures were approved by Shenzhen Institutes of Advanced Technology, Chinese Academy of Sciences Animal Care and Use Committee. Six‐to‐eight‐week‐old female BALB/c athymic nude mice were maintained under aseptic conditions in a small animal isolator and were housed in standard cages at 12 hour/12 hour light/dark cycle with free access to food and water. All animals were acclimated to the animal facility for at least 3 days before experimentation. All possible parameters that may cause social stress, like group size, type (treated and non‐treated), etc., among the experimental animals were carefully monitored and avoided. Tumour models were established by subcutaneous injection of 5 × 10^6^ U87 cells into the right flank of each mouse. Animals were observed daily for any behavioural abnormalities and weighed every 3 days. Tumour‐bearing mice were used when the volume of tumours reached to 100 mm^3^. BALB/C athymic nude mice orthotopic glioma models were established as follows. Animals were anesthetized intraperitoneally with chloral hydrate (40 mg/kg). After being immobilized in a stereotaxic apparatus (RWD68003; RWD Life Science Co, Shenzhen, China), the dorsal surface of the skull was sterilized with an iodine swab. A linear skin incision was placed over the bregma and a burr hole was drilled into the skull approximately 1.8 mm lateral and 1 mm posterior to the bregma. A 25 μL gas‐tight syringe was used to inject 5 × 10^5^ U87 cells suspended in 5 μL DMEM into the right frontal lobe at a depth of 3.5 mm relative to the dural surface of the brain. The cell suspension was slowly injected over 10 minutes. And then, the needle was slowly retracted over an additional 5 minutes. The wound was rinsed with 0.9% NaCl solution and the burr hole occluded with sterile bone wax to prevent leakage of the cerebrospinal fluid. The skin was then closed with absorbable sutures and the mice were allowed to recover from anaesthesia under observation. Each animal was given a one‐time dose by IP administration of antibiotics. MR images of the brain were acquired 7 days following implantation.

### 2.10 In vivo imaging

For in vivo imaging, mice with subcutaneous tumours and orthotopic glioma were used as the animal model. The fluorescence (FL) imaging experiments were performed when tumours grow to the right size. DVDMS‐Mn‐LPs or free DVDMS (DVDMS = 10 mg/kg) were intravenously (i.v.) injected into mice (n = 3), and fluorescence (FL) images were acquired on IVIS Spectrum Software (PerkinElmer) using a 430 nm excitation wavelength and a 680 nm filter at different time points. All mice were euthanized after the 24 hour imaging. Tumours as well as major organs were collected and subjected to ex vivo imaging. MRI imaging experiments were performed using a 3.0 T clinical MR scanner (TIM TRIO) with a small animal coil. After acquiring T_1_‐weighted MR images, the signal intensity was measured within a manually drawn region of interest for each mouse. The results were processed with intensity normalization and histogram matching.

### 2.11 In vivo SDT

Thirty mice‐bearing subcutaneous tumours were randomly divided into six groups as in vitro. The control group received no treatment, and the liposome group was intravenously injected DVDMS‐Mn‐LPs (DVDMS = 10 mg/kg) into mice. The U.S. group was irradiated by ultrasound with intensity of 1.5 W/cm^2^ for 10 minutes. The SDT group was treated intravenously with DVDMS‐Mn‐LPs and ultrasound of 1.5 W/cm^2^ for 10 minutes, while the mice of SDT plus skull and PDT plus skull groups were treated with SDT/PDT when the skull was covered on the subcutaneous tumour. The ultrasound or laser irradiation with intensity of 1.5 W/cm^2^ for 10 minutes was applied at 6 hour after the injection of DVDMS‐Mn‐LPs into mice. The tumour sizes and body weights were examined every 3 days for 30 days. Mice with tumour sizes over 1000 cm^3^ would be euthanatized according to animal protocol. The tumour volume was estimated using the formula, tumour volume = length × (width)^2^/2. The volume of tumour was evaluated by normalizing the measured values. In order to further verify the anti‐tumour effects of SDT, 18 mice bearing orthotopic glioma were randomly divided into three groups: the control group, the SDT group and the PDT group. The treatment strategies of each group were the same as subcutaneous tumour. MRI images of the brain were acquired at different times to monitor tumour growth. Animals were followed until death, up to 33 days. Total survival times from tumour implantation until death were recorded.

### 2.12 Histological analysis

After therapy, the mice were sacrificed by standard decapitation, and the tumours and major organs (heart, liver, spleen, lung and kidney) were harvested, fixed with 10% formalin and embedded in paraffin. 7‐μm sections were cut with a paraffin slicing machine, followed by staining with hematoxylin‐eosin (H&E) dyes. Tumour apoptosis was assessed by TUNEL assay according to the product instruction. Tumor proliferation potential was also assessed by immunohistochemistry staining through detecting the expression level of PCNA and Ki67, which were representative markers of tumour cell proliferation. The images were acquired on a biological inverted microscope (Olympus IX71).

### 2.13 Statistical analysis

Statistical analysis was carried out with SPSS version 19.0. All of the data represent mean values ± standard deviation of independent measurements. Statistical analysis was performed with a one way ANOVA. Differences were considered significant at *P < 0.05 and very significant at **P < 0.01.

## 3 RESULTS

### 3.1 Synthesis and characterization of DVDMS‐Mn‐LPs

**Figure 1:**
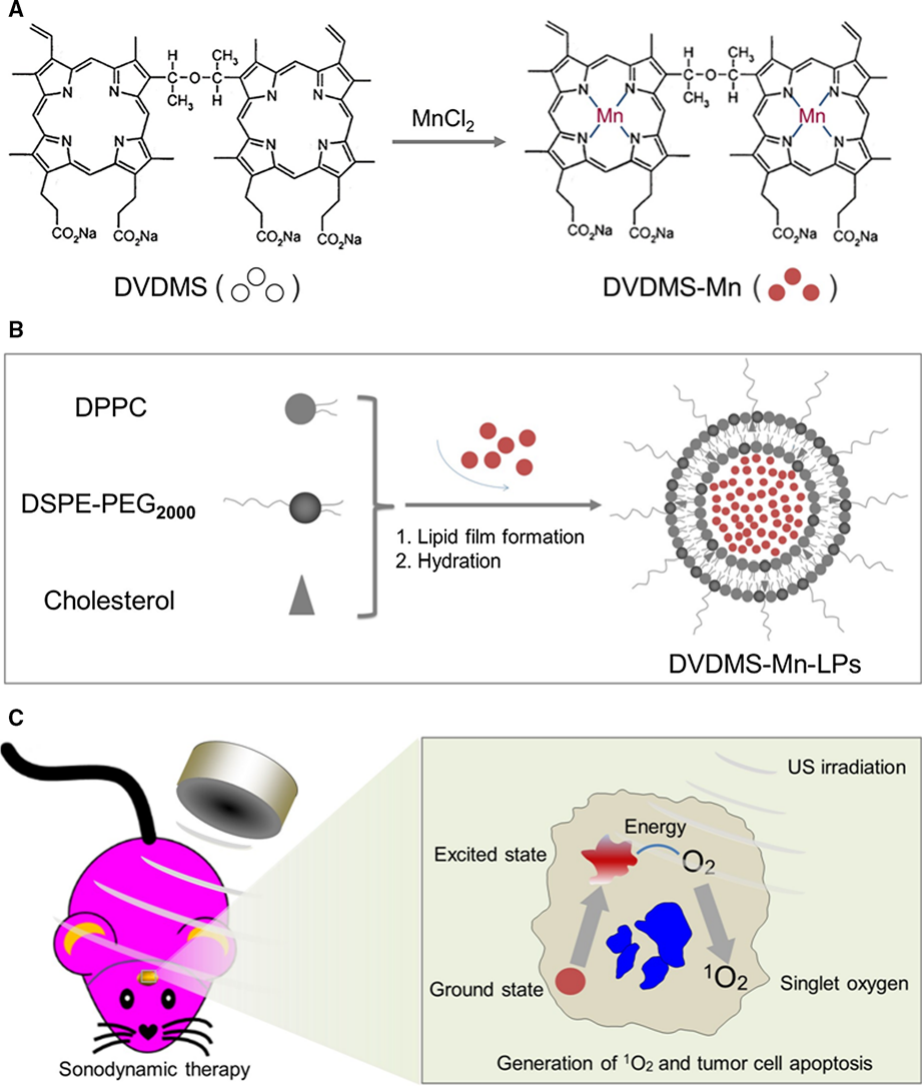
Preparation of DVDMS‐Mn‐LPs and mechanisms of sonodynamic therapy (SDT) with DVDMS‐Mn‐LPs. A, Schematic diagram of synthesis of DVDMS‐Mn. B, Schematic diagram of synthesis of DVDMS‐Mn‐LPs. C, The scheme of SDT of glioma seated into the brain and its mechanism to induce tumour cell apoptosis. DVDMS‐Mn‐LPs can be up taken by the tumour cells. Upon receiving US, DVDMS‐Mn‐LPs in the tumour cells would receive the US energy and be activated from a ground‐state into an excited state. When the excited‐stated DVDMS‐Mn‐LPs return to the ground‐stated, the energy could be released; O_2_ can absorb the released energy and change into ^1^O_2_. The ^1^O_2_ would cause the apoptosis and necrosis of tumour cells

**Figure 2:**
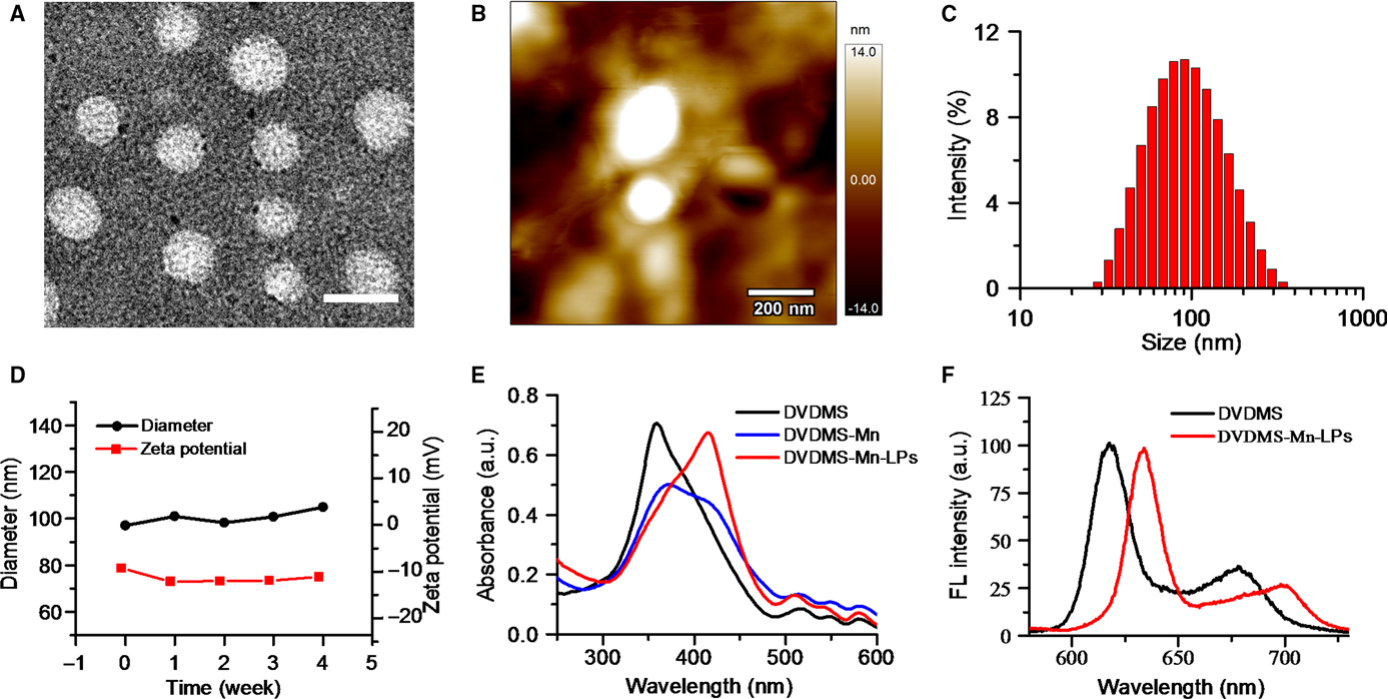
The characterization of DVDMS‐Mn‐LPs. A, TEM image of DVDMS‐Mn‐LPs (Scale bar: 200 nm). B, AFM image of DVDMS‐Mn‐LPs. C, Size distribution of DVDMS‐Mn‐LPs by dynamic light scattering. D, Stability of DVDMS‐Mn‐LPs, which were assessed by determining the average diameter and zeta potential at predetermined times. E, UV–vis absorption spectra of free DVDMS, DVDMS‐Mn and DVDMS‐Mn‐LPs. F, Fluorescence spectra of free DVDMS and DVDMS‐Mn‐LPs

**Figure 3:**
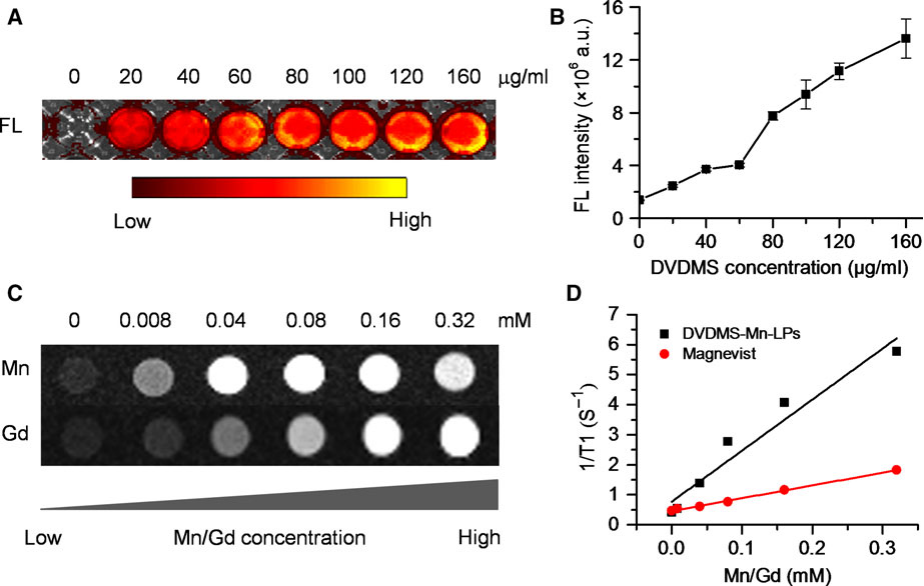
Imaging effects of DVDMS‐Mn‐LPs in vitro. A, Fluorescence images of DVDMS‐Mn‐LPs at different DVDMS concentrations. B, Fluorescence intensity of (A). The fluorescence intensity was recorded as photons per second per centimetre squared per steradian (p/s/cm^2^/sr). Data are presented as the mean ± standard deviation of three independent experiments. C, T_1_‐weighted MR images of DVDMS‐Mn‐LPs and Magnevist at different Mn or Gd concentrations. D, T_1_ relaxation rate of DVDMS‐Mn‐LPs and Magnevist measured at different Mn^2+^ or Gd^3+^ concentrations. DVDMS‐Mn‐LPs showed about four times higher than that of Magnevist, a clinically used agent

**Figure 4:**
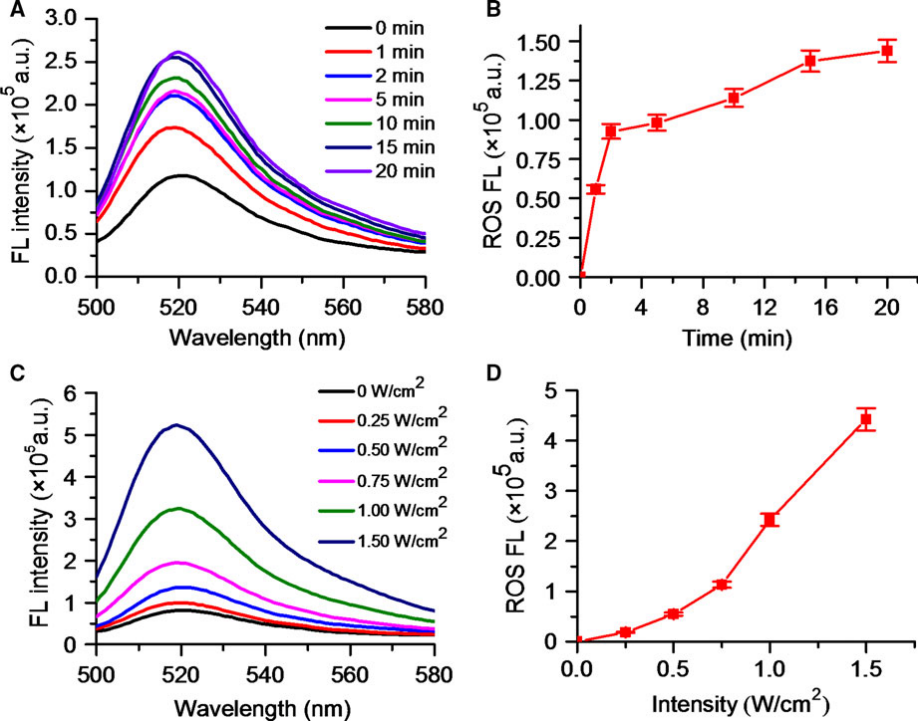
The ROS generation of DVDMS‐Mn‐LPs under US irradiation. A, Fluorescence emission spectra of DVDMS‐Mn‐LPs in DCFH‐DA solution with different US irradiation time. B, The fluorescence intensity changes of DCFH‐DA at 521 nm with the increase of US irradiation time. C, Fluorescence emission spectra of DVDMS‐Mn‐LPs in DCFH‐DA solution with different US irradiation intensities. D, The fluorescence intensity changes of DCFH‐DA at 521 nm with the increase of US irradiation intensity. DCFH‐DA, 2′,7′‐dichlorofluorescin diacetate; ROS, reactive oxygen species

As shown in Figure 1A presented the synthesis process of DVDMS‐Mn by chelating MnCl_2_ to DVDMS. Inductively coupled plasma atomic emission spectroscopy (ICP‐AES) measurement revealed that the chelation of Mn^2+^ with DVDMS occurred at a molar ratio of 1.6:1 after removal of unbound Mn^2+^ through dialysis. Then, DVDMS‐Mn was encapsulated into liposomes by thin‐film rehydration method, resulting in DVDMS‐Mn‐LPs as shown in Figure 1B. The encapsulation for these DVDMS‐Mn into the liposomes will produce the nanoparticles with the size about 100 nm, which favour their enrichment in the tumours. Also, it will decrease the potential cytotoxicity of DVDMS‐Mn. Transmission electron microscopy revealed that the resulting DVDMS‐Mn‐LPs exhibited a well‐defined spherical morphology and homogeneous distribution (Figure 2A). Meanwhile, atomic force microscope showed that the size of DVDMS‐Mn‐LPs was about 100 nm and the thickness was about 30 nm (Figures 2B and S2). Similar particle size was also obtained by dynamic light scattering (DLS) measurement (Figure 2C). The average diameter of DVDMS‐Mn‐LPs was 85.15 ± 2.14 nm with a polydispersity index (PDI) of 0.21 ± 0.019. Quantitative analysis revealed about 65.56 ± 1.47% DVDMS‐Mn was encapsulated into these nanoparticles. It was found that DVDMS‐Mn‐LPs had excellent stability, showing almost constant diameter size and polydispersity index for 4 weeks in PBS (Figure 2D). Figure 2E presented the optical absorption spectra of free DVDMS, DVDMS‐Mn and DVDMS‐Mn‐LPs. All of them exhibited strong Soret absorption at 359 nm, and Q‐bands between 500 and 600 nm. Fluorescence spectra had shown that the fluorescence emission peaks from DVDMS at 618 and 680 nm were shifted to 634 and 701 nm in DVDMS‐Mn‐LPs (Figure 2F). The fluorescence intensities progressively increased with the elevated concentrations of DVDMS‐Mn‐LPs from 20 to 160 μg/mL, showing a good linear variation for the fluorescence of DVDMS‐Mn‐LPs (Figure 3A,B). Figure 3C showed T_1_‐weighted MR images with the incremental signal intensity in the range of Mn^2+^ or Gd^3+^ ion (Magnevist) concentrations from 0.008 to 0.32 mmol/L. The T_1_ relativity coefficient (*r*_1_) value for DVDMS‐Mn‐LPs was 17.02 per mmol/L per seconds, which was about 4‐fold higher than that of the clinical approved Magnevist with 4.25 per mmol/L per seconds (Figure 3D). The singlet oxygen (^1^O_2_) could be generated from DVDMS‐Mn‐LPs dependent on U.S. time and intensity (Figure 4).

### 3.2 Cellular uptake and in vitro SDT

**Figure 5:**
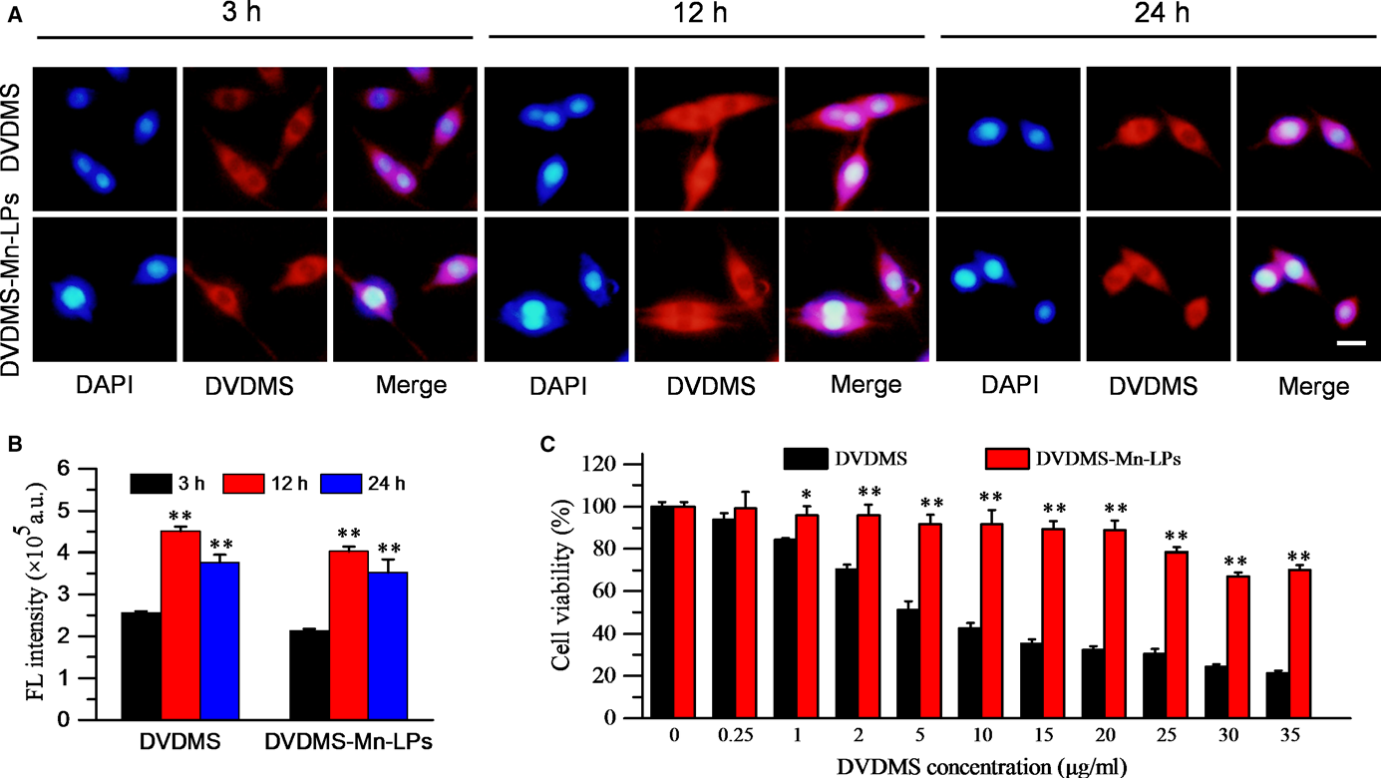
Cell uptake and cytotoxicity of free DVDMS and DVDMS‐Mn‐LPs. A, Fluorescence images displayed cellular uptake of free DVDMS and DVDMS‐Mn‐LPs after 3, 12 or 24 h incubation. Blue represented the fluorescence of DAPI and red represented the fluorescence of DVDMS (Scale bar: 20 μm). B, Geometric mean fluorescence intensity of free DVDMS and DVDMS‐Mn‐LPs from flow cytometric analysis at different incubation times (n = 3). C, Relative viability of U87 cells incubated with different concentrations of free DVDMS or DVDMS‐Mn‐LPs for 24 h. Cell viability was measured by the standard CCK8 assay. The data were shown as mean ± SD; *P < 0.05, **P < 0.01. CCK8, Cell Counting Kit 8; DAPI, 4′,6‐diamidino‐2‐phenylindole

The cell uptake behaviour of DVDMS‐Mn‐LPs was investigated on U87 cancer cells through fluorescence microscopy and flow cytometry. As shown in Figure 5A,B, the fluorescence intensities of cells received with DVDMS‐Mn‐LPs, similar with free DVDMS, gradually enhanced and achieved a peak at 12 hour. Biocompatibility assay by the CCK‐8 protocol revealed that DVDMS‐Mn‐LPs without ultrasound irradiation exhibited a significantly lower cytotoxicity to U87 cells than that of free DVDMS at various tested concentrations (>1 μg/mL; Figure 5C).

**Figure 6:**
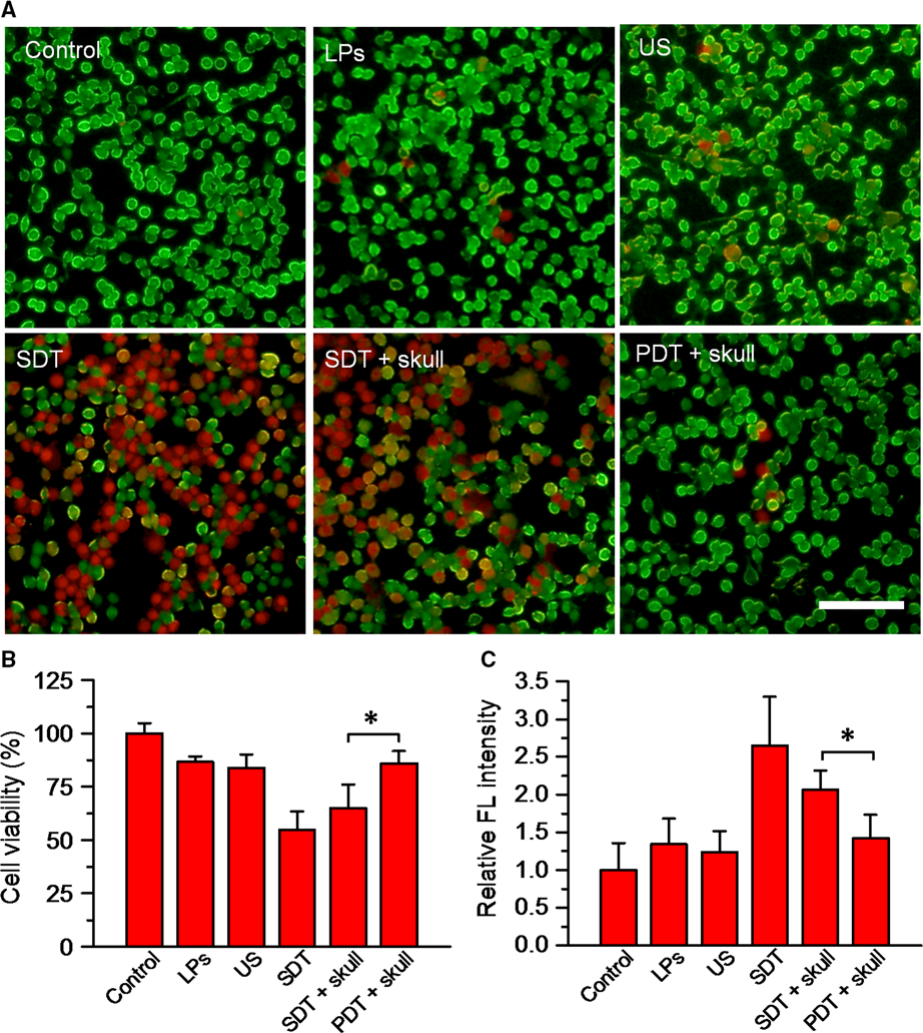
In vitro SDT of tumor cells. A, Fluorescence images of U87 cells after different treatments. Viable cells were stained green with calcein‐AM, and dead/later apoptosis cells were stained red with PI. The concentration of DVDMS = 20 μg/mL. LPs stand for DVDMS‐Mn‐LPs. SDT: Cells were treated with DVDMS‐Mn‐LPs and received with ultrasound irradiation. PDT: Cells were treated with DVDMS‐Mn‐LPs and received with laser irradiation (Scale bar: 100 μm). B, Quantitative evaluation of cell survivals of each group. C, The singlet oxygen levels of U87 cells, showing significantly higher level of SDT plus skull group than PDT plus skull group. The data were shown as mean ± SD; *P < 0.05. PDT, photodynamic therapy; SDT, sonodynamic therapy

To determine the therapeutic effect of SDT assisted by DVDMS‐Mn‐LPs, U87 cells were incubated with DVDMS‐Mn‐LPs for 3 hour, followed by ultrasound irradiation. The staining results by Calcein AM for the live cells and propidium iodide for the dead cells were shown in Figure 6A. Obviously, most of U87 cells receiving with either DVDMS‐Mn‐LPs or only ultrasound treatment displayed green fluorescence, indicating that both treatments could not kill cancer cells. In contrast, DVDMS‐Mn‐LPs plus ultrasound treatment displayed significant cell death (red fluorescence). Addition of a mouse skull on the beam path did not significantly decrease the SDT effect of DVDMS‐Mn‐LPs plus ultrasound, but really decreased the PDT effect of DVDMS‐Mn‐LPs. The cell viability was quantitatively assessed by CCK‐8 kit, revealing that incubation with DVDMS‐Mn‐LPs or only ultrasound irradiation treatment could only induce small partial cell death, with 86.54 ± 2.67% or 83.76 ± 6.48% cell viability, respectively. In marked contrast, the combination treatments, regardless of the SDT and SDT plus skull, were found to be highly effective in destructing cancer cells, resulting in 54.82 ± 8.55% or 64.91 ± 11.02% cell viability, respectively. As expected, the PDT plus skull group showed 85.95 ± 5.60% cell viability (Figure 6B). The levels of intracellular ROS detected by ROS probe further revealed the cytotoxicity mechanism of SDT. As shown in Figure 6C, the tumour cells treated with SDT caused a significantly increased level of ROS, with 1.98‐fold, 2.15‐fold and 2.65‐fold higher than that of the tumour cells treated with only DVDMS‐Mn‐LPs, only ultrasound and PBS control, respectively. Comparatively, the treatment with PDT plus skull produced much less ROS than that of SDT plus skull.

### 3.3 Dual‐modal imaging in vivo

**Figure 7:**
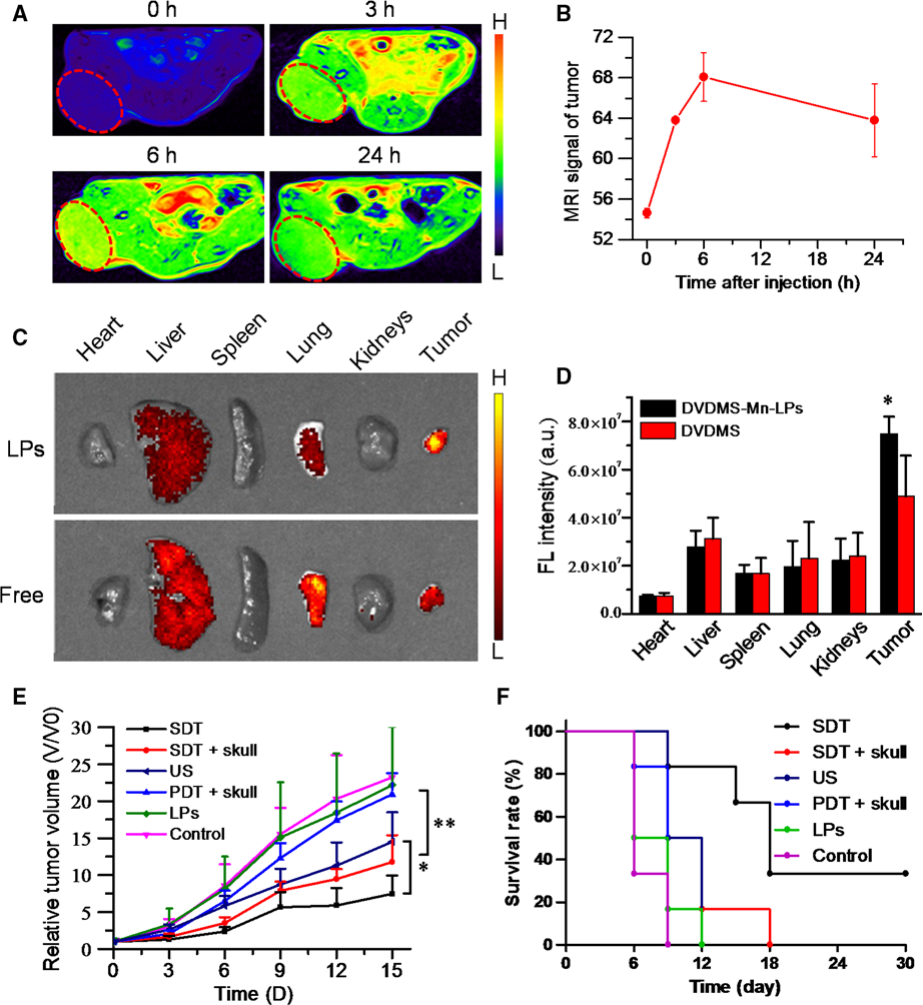
In vivo SDT of U87 subcutaneous tumour. A, In vivo MR imaging of subcutaneous tumour‐bearing mice taken at different time points post i.v. injection of DVDMS‐Mn‐LPs at the same dose (DVDMS = 10 mg/kg). B, The quantification of average T_1_ MR signals (Contrast‐to‐Noise ratio, CNR) in the tumour at different post‐injection time. C, Ex‐vivo fluorescence images of major organs and tumours were obtained at 24 h post‐injection of free DVDMS or DVDMS‐Mn‐LPs. D, The quantitative analysis of fluorescence intensity for these organs and tumours, revealing higher signal intensity in the tumour of mice received with DVDMS‐Mn‐LPs than that of received with free DVDMS. E, Tumour growth curves of U87 tumour‐bearing mice from different treatments. F, Survival rates of mice of these tumour‐bearing mice after different treatments. The data were shown as mean ± SD; *P < 0.05, **P < 0.01. SDT, sonodynamic therapy

The cross‐section T_1_‐weighted MR images were acquired at different time intervals after intravenous injection of DVDMS‐Mn‐LPs into subcutaneous tumour‐bearing mice (Figure 7A). The brighter T_1_ MR signals in the tumour appeared at the third hour and gradually reached the plateau after 6 hour (Figure 7B). The in vivo T_1_‐weighted MR imaging of orthotopic glioma‐bearing mice was also carried out. The T_1_ signals of tumour on mice strengthened with the increase in time interval, and reached a peak after 3 hour injection of DVDMS‐Mn‐LPs (Figure S3).

In vivo fluorescence imaging was performed on IVIS Spectrum system using a 430 nm excitation wavelength and a 680 nm filter at different time points after injection of free DVDMS or DVDMS‐Mn‐LPs. As shown in Figure S4A, free DVDMS showed a relatively weak fluorescence in orthotopic glioma. After 24 hour post‐injection, almost no fluorescence signal in the brain was observed. Comparatively, after intravenous injection of DVDMS‐Mn‐LPs, the fluorescence signal of orthotopic glioma could be clearly distinguished from surrounding normal tissue after 3 hour post‐injection, and maintained at this level till 12 hour (Figure S4). Those mice were then sacrificed at 24 hour intravenous injection, with major organs taken for ex vivo fluorescent imaging. The biodistribution of nanoparticles was mainly located in the liver, lung and tumour. By comparison, the fluorescence signal intensity of DVDMS‐Mn‐LPs accumulated in tumour was stronger than that of free DVDMS (Figure 7C,D).

### 3.4 Imaging‐guided in vivo SDT

The SDT effect in vivo was shown in Figure 7E,F, for tumours receiving DVDMS‐Mn‐LPs or PDT plus skull treatments, their growths were not obviously inhibited, almost the same as the untreated control. Only ultrasound treatment produced partial tumour growth inhibition. Importantly, both the SDT treatment and SDT plus skull treatment with the assistance of DVDMS‐Mn‐LPs could inhibit the growth of tumours. No significant difference of the therapeutic efficacies was found between the SDT and SDT plus skull groups. A significantly longer survival period was also observed for the groups treated with SDT and SDT plus skull than that of the other groups (*P* < 0.01). Moreover, neither obvious body weight loss nor pathological damage was found in the six groups of mice (Figures S5 and S6), indicating no significant acute toxicity for the theranostic agent.

**Figure 8:**
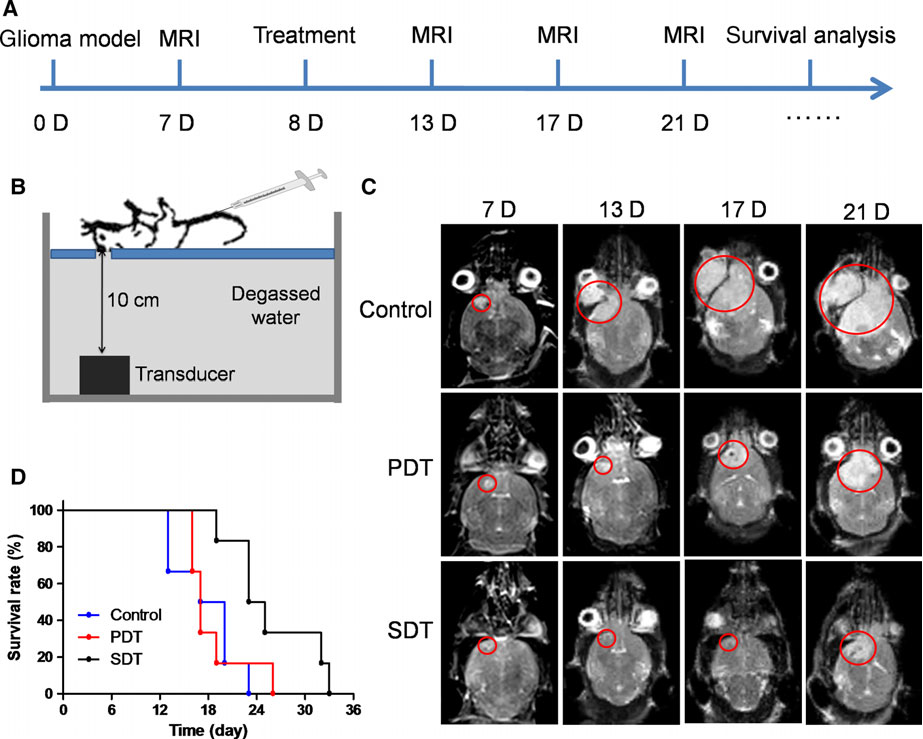
In vivo SDT of orthotopic glioma. A, Flow chart of SDT procedure for orthotopic glioma. B, Schematic diagram of the insonation device in vivo. C, MR images of different groups of orthotopic U87 tumours at different times. D, Survival rates of orthotopic tumour‐bearing mice after different treatments. SDT, sonodynamic therapy

To further examine the treatment efficacy against orthotopic glioma, DVDMS‐Mn‐LPs were intravenously administrated to the orthotopic glioma mouse model followed by SDT process. The experimental scheme over time was shown in Figure 8A and the experimental setting was setup as Figure 8B. The growth of orthotopic glioma received SDT was obviously inhibited and the survival time of tumour‐bearing mice was significantly extended in comparison with PDT (Figure 8C,D).

Hematoxylin and eosin (HE) staining and terminal deoxynucleotidyl transferase dUTP nick end labelling (TUNEL) assays were utilized to examine the morphology and apoptosis of tumour cells. In terms of the control, DVDMS‐Mn‐LPs and PDT plus skull groups, the tumour cells still retained their normal morphology with apparent membrane and nuclear structure, but the tumour in the SDT and SDT plus skull groups showed apparent necrosis. Compared with PDT, SDT, and SDT plus skull groups showed much more apoptotic cancer cells. Immunohistochemistry staining assay demonstrated that PCNA and Ki67 were highly expressed in the control, DVDMS‐Mn‐LPs and PDT plus skull groups, but significantly decreased in the SDT and SDT plus skull groups (Figure S7).

## 4 DISCUSSION

Since porphyrin and its derivatives can capture metal ions as chelating molecules,^26^, ^31^, ^32^ we speculated that DVDMS, a porphyrin‐based photosensitizer, could form a chelate with manganese‐II (Mn^2+^), which confirmed by ICP‐AES measurement. To improve the stability and biocompatibility, DVDMS‐Mn was encapsulated into liposomes by thin‐film rehydration method (Figure 1A,B). Encapsulation of DVDMS‐Mn into lipids would help them accumulate into glioma due to the leaky endothelium in the tumour vasculature.^33^ It is important since ultrasound need to penetrate through the skull to activate these sonosensizers from ground state to excited state, by which ultrasound energy is transmitted to O_2_ and generate ^1^O_2_ to induce tumour cell apoptosis (Figure 1C). Figure 2 had shown that DVDMS‐Mn‐LPs are physiologically stable and biologically compatible. A red‐shifted absorption peak could be observed in the DVDMS‐Mn‐LPs, suggesting successful loading of DVDMS‐Mn into the nanoparticles (Figure 2E,F). The red‐shift of the absorption peak may be attributed to the formation of porphyrin aggregates and the possible interaction of the two sterically hindered porphyrin rings in DVDMS.^24^

The optical and T_1_ relaxation properties of DVDMS‐Mn‐LPs hold promises for dual‐mode contrast agent for fluorescence and MR imaging of tumours (Figure 3). In fact, abundant macromolecules and nanoparticles have been used as platforms for manganese loading, resulting in improved *r*_1_ value of chelated Mn^2+^ (generally between 5 and 30 per mmol/L per seconds).^27^, ^34^, ^35^, ^36^, ^37^ In our study, the *r*_1_ value for DVDMS‐Mn‐LPs was about 4‐fold higher than that of the clinical approved Magnevist due to increased local concentration of Mn^2+^ and lowered molecular tumbling rate in those nanocarrier systems.^34^, ^35^, ^36^, ^37^ Given that the generation of ROS plays an important role in SDT of tumour treatment, the potential of DVDMS‐Mn‐LPs to generate ROS was determined using 2′, 7′‐dichlorofluorescin diacetate as a typical probe.^23^, ^38^ The probe will be deacetylated by cellular esterases to non‐fluorescent 2′,7′‐Dichlorodihydrofluorescin (DCFH), which is rapidly oxidized to highly fluorescent 2′,7′‐Dichlorodihydrofluorescein (DCF) by ROS. These results clearly revealed that the DVDMS‐Mn‐LPs could act as an effective SDT sonosensitizer for the generation of toxic ROS (Figure 4).

The low cytotoxicity is especially important for a SDT agent to guarantee its biological safety when it is intravenously injected into animal or human body before receiving ultrasound irradiation. Additionally, the previous reports have shown that free DVDMS had obvious cytotoxicity to some cells, even if at lower concentrations (0.5 μg/mL).^24^, ^29^ Our study provides a safer DVDMS formulation which has no obvious cell damage below 20 μg/mL DVDMS concentration before they receive laser or ultrasound irradiation (Figure 5). Although a large amount of literatures available suggested that PDT has promising applications in superficial tumours, our results imply that PDT is much less effective in the treatment of tumours seated in deep tissues while SDT is featured with high efficiency for solving this critical issue, especially in the treatment of brain tumours. Because low frequency ultrasound has a strong penetrability through the mouse skull and focus into the brain in a small volume, achieving a precise position on tumours.^18^, ^19^, ^20^, ^21^

DVDMS‐Mn‐LPs not only could be used as dual‐modal contrast agents for MRI and fluorescence imaging in vitro but also in vivo. Free DVDMS could be quickly excreted with a low tumor accumulation amount, but DVDMS‐Mn‐LPs accumulated in tumour effectively (Figure 7). The reasons may be attributable to the passive tumour targeting by enhanced permeability and retention effect. In fact, the previous reports had demonstrated that nanoparticles could permeate into brain tumours due to the destruction of blood brain barrier.^39^, ^40^ All these results showed that the DVDMS‐Mn‐LPs successfully integrated two imaging modalities into a single nanoscale system, which provided the basis for further imaging‐guided treatment experiments.

With the assistance of imaging guidance, the ultrasound beam could be precisely and flexibly positioned to the whole tumour. Both the SDT treatment and SDT plus skull treatment could inhibit the growth of tumours, supported that SDT but not PDT has greater anti‐tumour efficacy for glioma treatment. The SDT and SDT plus skull groups had no significant difference of the therapeutic efficacies, suggesting that the low frequency ultrasound has a strong penetrability through the mouse skull. All these results indicate that the SDT with DVDMS‐Mn‐LPs is a safe and effective glioma treatment technique.

## 5 CONCLUSIONS

In summary, we have successfully developed, for the first time, a multifunctional theranostic nano‐sonosensitizers (DVDMS‐Mn‐LPs) for highly efficient imaging‐guided SDT against glioma, as systematically demonstrated both in vitro and in vivo. The DVDMS‐Mn‐LPs nanosensitizers fully integrate the specific advantages of porphyrin‐based organic sonosensitizers for efficient SDT and Mn^2+^ chelating for contrast‐enhanced T_1_‐weighted MR imaging based on metalloporphyrin chemistry. The *r*_1_ relaxivity of the DVDMS‐Mn‐LPs has been found to be 4 times higher than that of traditional clinical‐used and Gd‐based Magnevist, providing the potential application value for imaging‐guided SDT. SDT assisted by DVDMS‐Mn‐LPs significantly enhanced the anticancer effect than that of PDT with DVDMS‐Mn‐LPs in the presence of skull because of the high tissue‐penetrating capability of ultrasound as the irradiation source. This nanoparticle‐assisted SDT modality on combating cancer paves a totally new way for the glioma treatment by breaking the depth shadow of traditional photoactivation (PDT) based on the unique characteristics of SDT with non‐invasiveness and high penetrability.

## CONFLICT OF INTEREST

The authors declare no conflicts of interest.

## AUTHOR CONTRIBUTION

H.L. and M.Z. contributed equally. F.Y. and H.Z. conceived and designed the experiments. H.L., M.Z., Z.S., W.C. and J.L. performed the experiments. F.Y. and M.Z. analysed the data and wrote the paper. All authors discussed the results and commented on the manuscript.

## References

[1] Wen PY , Kesari S . Malignant gliomas in adults. N Engl J Med. 2008;359(5):492‐507. 18669428 10.1056/NEJMra0708126

[2] Alifieris C , Trafalis DT . Glioblastoma multiforme: pathogenesis and treatment. Pharmacol Ther. 2015;152:63‐82. 25944528 10.1016/j.pharmthera.2015.05.005

[3] Paw I , Carpenter RC , Watabe K , Debinski W , Lo H‐W . Mechanisms regulating glioma invasion. Cancer Lett. 2015;362(1):1‐7. 25796440 10.1016/j.canlet.2015.03.015 PMC4435977

[4] Wait SD , Prabhu RS , Burri SH , Atkins TG , Asher AL . Polymeric drug delivery for the treatment of glioblastoma. Neuro Oncol. 2015;17:ii9‐ii23. 25746091 10.1093/neuonc/nou360 PMC4483039

[5] Gallego PJ , Delattre JY . Management of elderly patients with gliomas. Oncologist. 2014;19(12):1258‐1267. 25342314 10.1634/theoncologist.2014-0170 PMC4257742

[6] Juratli TA , Schackert G , Krex D . Current status of local therapy in malignant gliomas–a clinical review of three selected approaches. Pharmacol Ther. 2013;139(3):341‐358. 23694764 10.1016/j.pharmthera.2013.05.003

[7] Chu KF , Dupuy DE . Thermal ablation of tumours: biological mechanisms and advances in therapy. Nat Rev Cancer. 2014;14(3):199‐208. 24561446 10.1038/nrc3672

[8] Shanmugam V , Selvakumar S , Yeh CS . Near‐infrared light‐responsive nanomaterials in cancer therapeutics. Chem Soc Rev. 2014;43(17):6254‐6287. 24811160 10.1039/c4cs00011k

[9] Zhang Z , Wang J , Chen C . Near‐infrared light‐mediated nanoplatforms for cancer thermo‐chemotherapy and optical imaging. Adv Mater. 2013;25(28):3869‐3880. 24048973 10.1002/adma.201301890

[10] Kim YS , Kim BG , Rhim H , et al. Uterine fibroids: semiquantitative perfusion MR imaging parameters associated with the intraprocedural and immediate postprocedural treatment efficiencies of MR imaging‐guided high‐intensity focused ultrasound ablation. Radiology. 2014;273(2):462‐471. 24988436 10.1148/radiol.14132719

[11] Lin C , Cheng Y , Chen M , Lin SM . The effectiveness of multiple electrode radiofrequency ablation in patients with hepatocellular carcinoma with lesions more than 3 cm in size and barcelona clinic liver cancer stage A to B2. Liver Cancer. 2016;5(1):8‐20. 26989656 10.1159/000367755 PMC4789949

[12] Ram Z , Cohen ZR , Harnof S , et al. Magnetic resonance imaging‐guided high‐intensity focused ultrasound for brain tumor therapy. Neurosurgery. 2006;59(5):949‐955. 17143231 10.1227/01.NEU.0000254439.02736.D8

[13] Chrastina J , Novak Z , Feitová V , Ríha I . Experience with radiofrequency termoablation of brain tumors. Rozhl Chir. 2008;87(7):338‐343. 18810924

[14] Dąbrowski JM , Arnaut LG . Photodynamic therapy (PDT) of cancer: from local to systemic treatment. Photochem Photobiol Sci. 2015;14(10):1765‐1780. 26219737 10.1039/c5pp00132c

[15] Yumita N , Nishigaki R , Umemura K , Umemura S . Hematoporphyrin as a sensitizer of cell‐damaging effect of ultrasound. Jpn J Cancer Res. 1989;80(3):219‐222. 2470713 10.1111/j.1349-7006.1989.tb02295.x PMC5917717

[16] Umemura S , Yumita N , Nishigaki R , Umemura K . Mechanism of cell damage by ultrasound in combination with hematoporphyrin. Jpn J Cancer Res. 1990;81(9):962‐966. 2172198 10.1111/j.1349-7006.1990.tb02674.x PMC5918111

[17] Umemura S , Yumita N , Nishigaki R . Enhancement of ultrasonically induced cell damage by a gallium‐porphyrin complex, ATX‐70. Jpn J Cancer Res. 1993;84(5):582‐588. 8320175 10.1111/j.1349-7006.1993.tb00179.x PMC5919169

[18] Trendowski M . The promise of sonodynamic therapy. Cancer Metastasis Rev. 2014;33(1):143‐160. 24346159 10.1007/s10555-013-9461-5

[19] Wang J , Guo Y , Gao J , et al. Detection and comparison of reactive oxygen species (ROS) generated by chlorophyllin metal (Fe, Mg and Cu) complexes under ultrasonic and visible‐light irradiation. Ultrason Sonochem. 2011;18(5):1028‐1034. 21236719 10.1016/j.ultsonch.2010.12.006

[20] Trendowski M . Using the promise of sonodynamic therapy in the clinical setting against disseminated cancers. Chemother Res Pract. 2015;2015:316015. 26380110 10.1155/2015/316015 PMC4562321

[21] Wood AK , Sehgal CM . A review of low‐intensity ultrasound for cancer therapy. Ultrasound Med Biol. 2015;41(4):905‐928. 25728459 10.1016/j.ultrasmedbio.2014.11.019 PMC4362523

[22] Yan F , Wu H , Liu H , et al. Molecular imaging‐guided photothermal/photodynamic therapy against tumor by iRGD‐modified indocyanine green nanoparticles. J Control Release. 2016;224:217‐228. 26739551 10.1016/j.jconrel.2015.12.050

[23] Hu D , Sheng Z , Gao G , et al. Activatable albumin‐photosensitizer nanoassemblies for triple‐modal imaging and thermal‐modulated photodynamic therapy of cancer. Biomaterials. 2016;93:10‐19. 27061266 10.1016/j.biomaterials.2016.03.037

[24] Yan X , Niu G , Lin J , et al. Enhanced fluorescence imaging guided photodynamic therapy of sinoporphyrin sodium loaded graphene oxide. Biomaterials. 2015;42:94‐102. 25542797 10.1016/j.biomaterials.2014.11.040 PMC4280503

[25] Gong H , Dong Z , Liu Y , et al. Engineering of multifunctional nano‐micelles for combined photothermal & photodynamic therapy under the guidance of multimodal imaging. Adv Funct Mater. 2014;24:6492‐6502.

[26] Chen Q , Wang X , Wang C , Feng L , Li Y , Liu Z . Drug‐induced self‐assembly of modified albumins as nano‐theranostics for tumor‐targeted combination therapy. ACS Nano. 2015;9(5):5223‐5233. 25950506 10.1021/acsnano.5b00640

[27] Huang P , Qian XQ , Chen Y , et al. Metalloporphyrin‐encapsulated biodegradable nanosystems for highly efficient magnetic resonance imaging‐guided sonodynamic cancer. J Am Chem Soc. 2017;139(3):1275‐1284. 28024395 10.1021/jacs.6b11846

[28] Wang X , Hu J , Wang P , et al. Analysis of the in vivo and in vitro effects of photodynamic therapy on breast cancer by using a sensitizer, sinoporphyrin sodium. Theranostics. 2015;5(7):772‐786. 25897341 10.7150/thno.10853 PMC4402500

[29] Xiong W , Wang P , Hu J , et al. A new sensitizer DVDMS combined with multiple focused ultrasound treatments: an effective antitumor strategy. Sci Rep. 2015;5:17485. 26631871 10.1038/srep17485 PMC4668354

[30] Liu Y , Wang P , Liu Q , Wang X . Sinoporphyrin sodium triggered sono‐photodynamic effects on breast cancer both in vitro and in vivo. Ultrason Sonochem. 2016;31:437‐448. 26964970 10.1016/j.ultsonch.2016.01.038

[31] MacDonald TD , Liu TW , Zheng G . An MRI‐sensitive, non‐photobleachable porphysome photothermal agent. Angew Chem Int Ed Engl. 2014;53(27):6956‐6959. 24840234 10.1002/anie.201400133

[32] Zhang XA , Lovejoy KS , Jasanoff A , Lippard SJ . Water‐soluble porphyrins as a dual‐function molecular imaging platform for MRI and fluorescence zinc sensing. Proc Natl Acad Sci U S A. 2007;104(26):10780‐10785. 17578918 10.1073/pnas.0702393104 PMC1904115

[33] Vieira DB , Gamarra LF . Getting into the brain: liposome‐based strategies for effective drug delivery across the blood‐brain barrier. Int J Nanomedicine. 2016;11:5381‐5414. 27799765 10.2147/IJN.S117210 PMC5077137

[34] Cui L , Lin Q , Jin CS , et al. A PEGylation‐free biomimetic porphyrin nanoplatform for personalized cancer theranostics. ACS Nano. 2015;9(4):4484‐4495. 25830219 10.1021/acsnano.5b01077

[35] Chen Q , Liang C , Wang C , Liu Z . An imagable and photothermal “Abraxane‐like” nanodrug for combination cancer therapy to treat subcutaneous and metastatic breast tumors. Adv Mater. 2015;27(5):903‐910. 25504416 10.1002/adma.201404308

[36] Jing L , Liang X , Li X , et al. Mn‐porphyrin conjugated Au nanoshells encapsulating doxorubicin for potential magnetic resonance imaging and light triggered synergistic therapy of cancer. Theranostics. 2014;4(9):858‐871. 25057312 10.7150/thno.8818 PMC4107288

[37] Liu R , Jing L , Peng D , Li Y , Tian J , Dai Z . Manganese (II) chelate functionalized copper sulfide nanoparticles for efficient magnetic resonance/photoacoustic dual‐modal imaging guided photothermal therapy. Theranostics. 2015;5(10):1144‐1153. 26284144 10.7150/thno.11754 PMC4533097

[38] Yan X , Hu H , Lin J , et al. Optical and photoacoustic dual‐modality imaging guided synergistic photodynamic/photothermal therapies. Nanoscale. 2015;7(6):2520‐2526. 25573051 10.1039/c4nr06868h PMC5257288

[39] Mangraviti A , Tzeng SY , Kozielski KL , et al. Polymeric nanoparticles for nonviral gene therapy extend brain tumor survival in vivo. ACS Nano. 2015;2:9. 10.1021/nn504905q PMC4342728 25643235

[40] Bertucci A , Prasetyanto EA , Septiadi D , et al. Combined delivery of temozolomide and anti‐miR221 PNA using mesoporous silica nanoparticles induces apoptosis in resistant glioma cells. Small. 2015;11(42):5687‐5695. 26395266 10.1002/smll.201500540

