# Supplementary material for: Theranostic nanosensitizers for highly efficient MR/fluorescence imaging‐guided sonodynamic therapy of gliomas
Source: J Cell Mol Med. 2018 Aug 29;22(11):5394–405. doi: 10.1111/jcmm.13811 (PMC6201228; doi:10.1111/jcmm.13811)
Supplement: Supplementary file 1 [file JCMM-22-5394-s001.doc]

Supporting Information

**Theranostic Nanosensitizers for Highly Efficient MR/Fluorescence Imaging-Guided Sonodynamic Therapy of Gliomas**

Hongmei Liu ^a, 1^, Meijun Zhou ^a, 1^, Zonghai Sheng ^b^, Yu Chen ^c^, Chih-Kuang ^d^, Wenting Chen ^e^, Jia Liu ^f^, Xin Liu ^b^, Fei Yan ^a, b, *^, Hairong Zheng ^a, b^

a. Department of Ultrasonography, Guangdong Second Provincial General Hospital, Guangzhou, China

b. Paul C. Lauterbur Research Center for Biomedical Imaging, Institute of Biomedical and Health Engineering, Shenzhen Institutes of Advanced Technology, Chinese Academy of Sciences, Shenzhen, China

c. State Key Laboratory of High Performance Ceramic and Superfine Microstructures, Shanghai Institute of Ceramics, Chinese Academy of Sciences, Shanghai, China

d. Department of Biomedical Engineering and Environmental Sciences, National Tsing Hua University, Taiwan

e. School of Life Science and Engineering, Southwest University of Science and Technology, Mianyang, China

f. Department of Ultrasonography, Guangdong Provincial Hospital of Chinese Medicine, The Second Clinical College of Guangzhou University of Chinese Medicine, Guangzhou, China

^1^ These authors contributed equally to this work.

*Corresponding authors at: Shenzhen Institutes of Advanced Technology, Chinese Academy of Sciences, Shenzhen, 518055, China.

E-mail address: [fei.yan@siat.ac.cn](mailto:hr.zheng@siat.ac.cn) (F. Yan). Tel: +86 755 86392284 Fax: +86 755 96382299


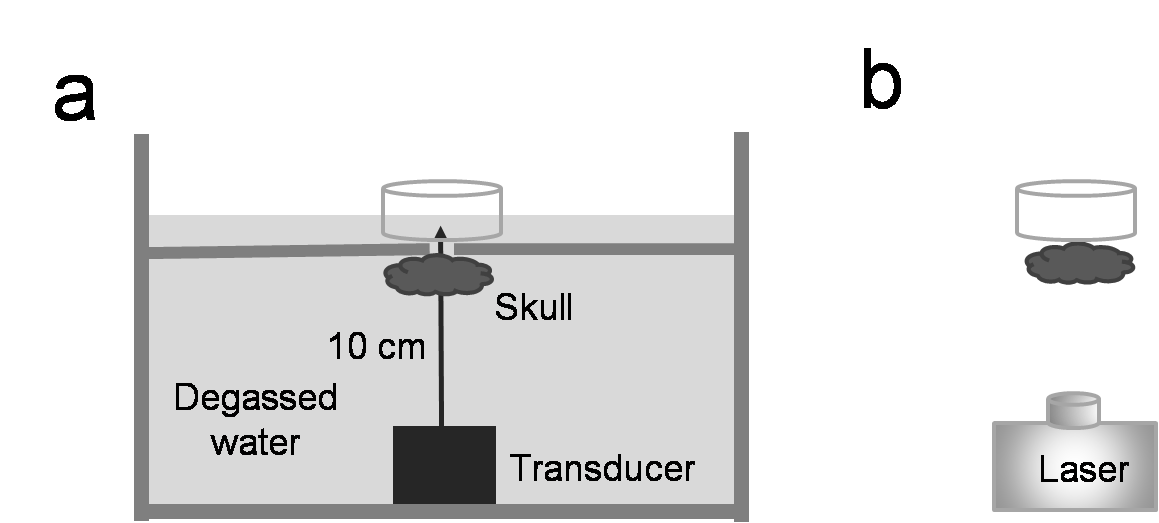


Figure S1. (a) Schematic diagram of the ultrasound device in vitro. (b) Schematic diagram of the laser device in vitro.


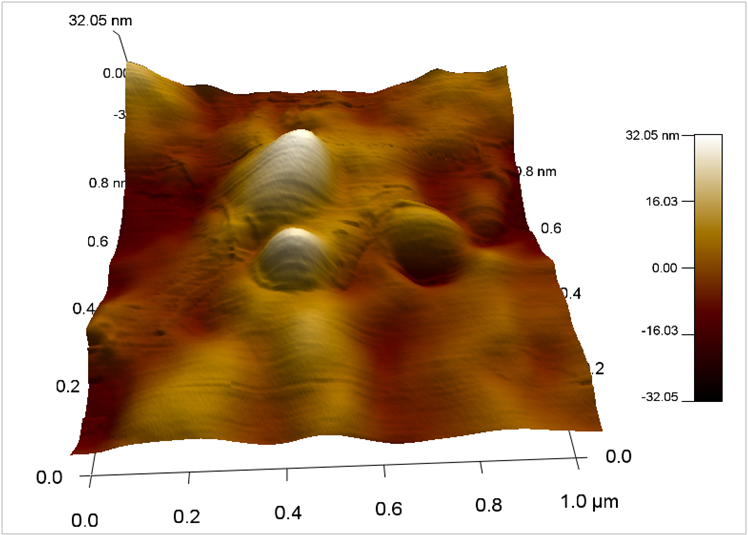


Figure S2. AFM image of DVDMS-Mn-LPs with a 3D view.


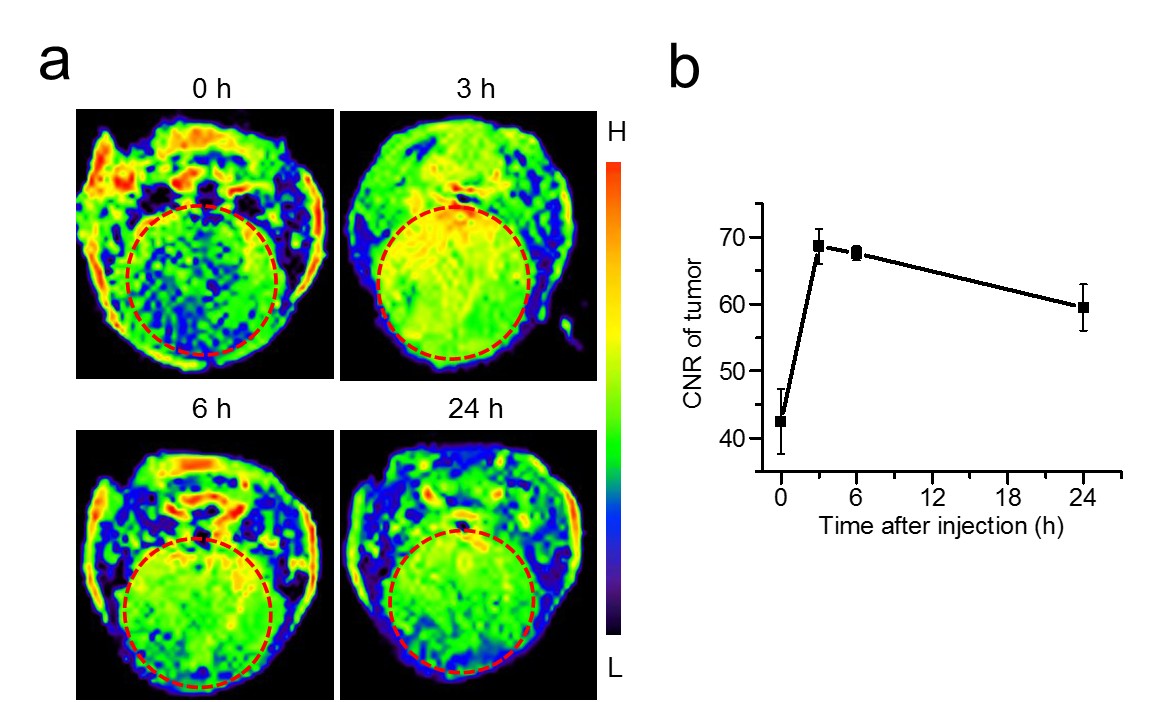


Figure S3. In vivo orthotopic glioma MR imaging. (a) In vivo MR imaging of orthotopic glioma-bearing mice taken at different time points post i.v. injection of DVDMS-Mn-LPs at the same dose (DVDMS = 10 mg/kg). (b) The quantification of average T_1_ MR signals (Contrast-to-Noise ratio, CNR) in the tumor regions at different post-injection time.


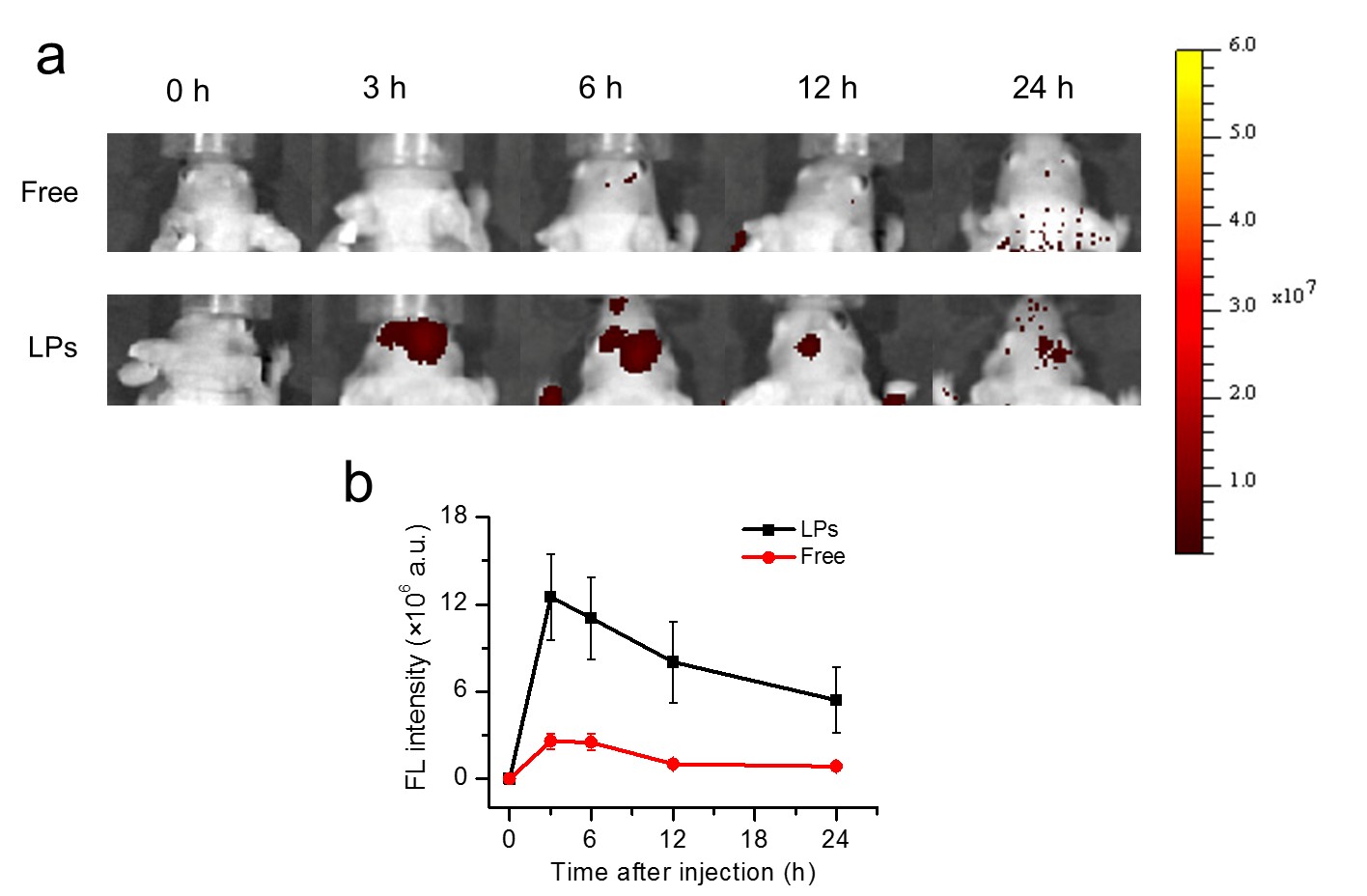


Figure S4. In vivo orthotopic glioma fluorescence imaging. (a) In vivo fluorescence images of orthotopic glioma-bearing mice taken at different time points post i.v. injection of DVDMS-Mn-LPs at the same dose (DVDMS = 10 mg/kg). (b) The mean fluorescence intensity of orthotopic glioma at 0, 3, 6, 12 and 24 h postinjection, respectively.


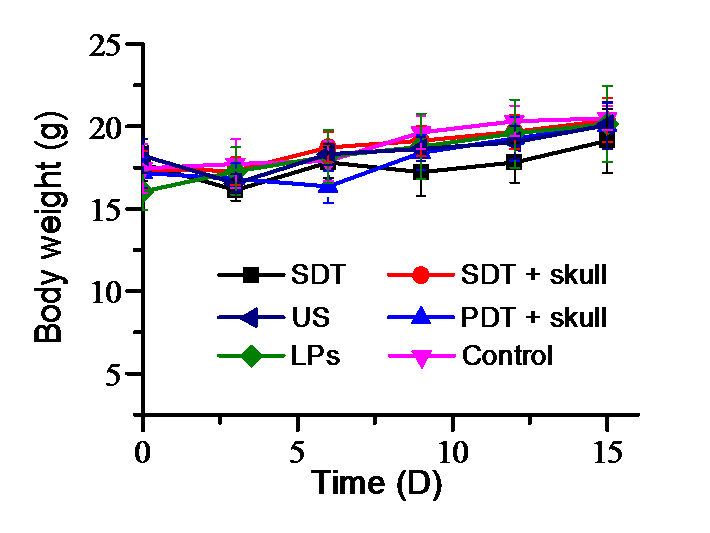


Figure S5. Body weights were measured during the 15 day evaluation period in mice under the different treatments.


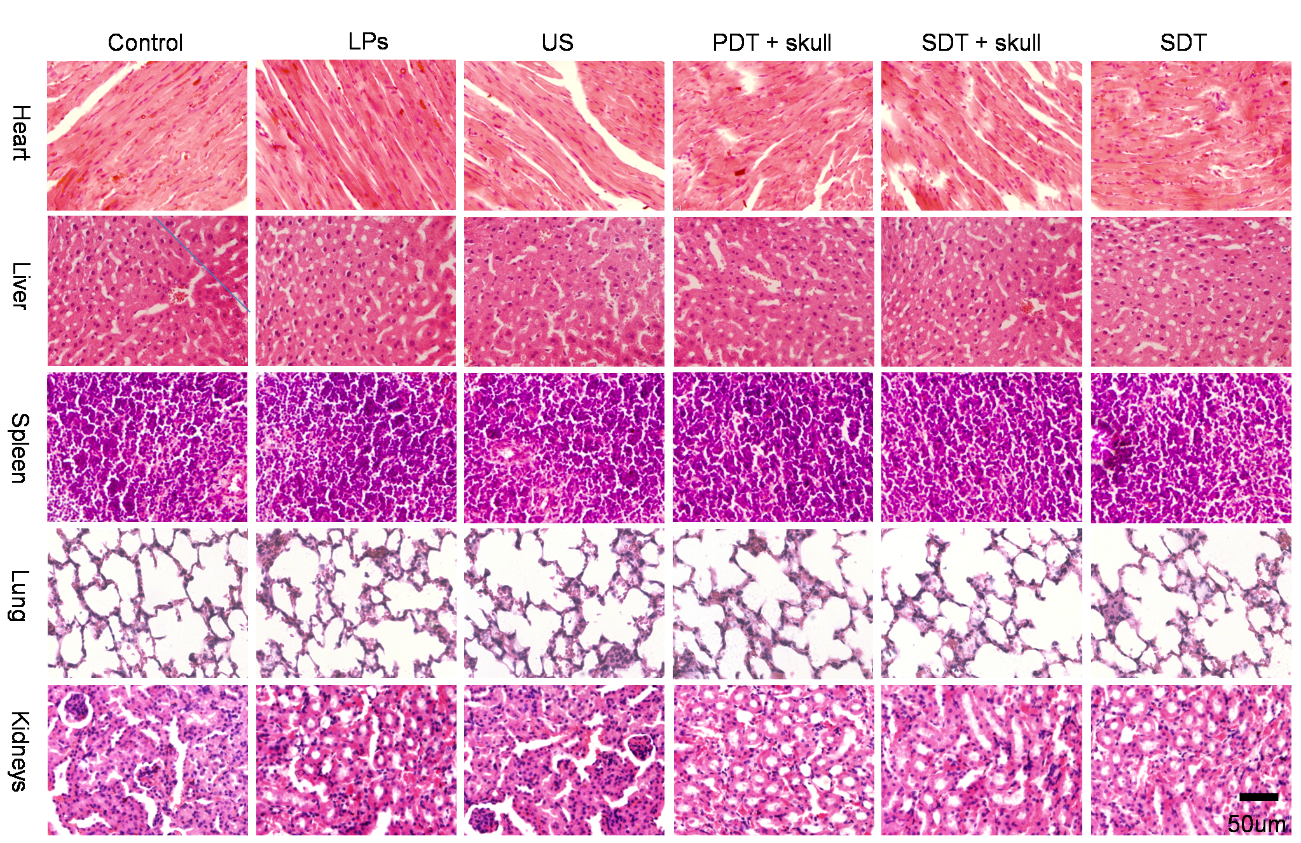


Figure S6. H&E staining images of major organs collected from different treated groups.


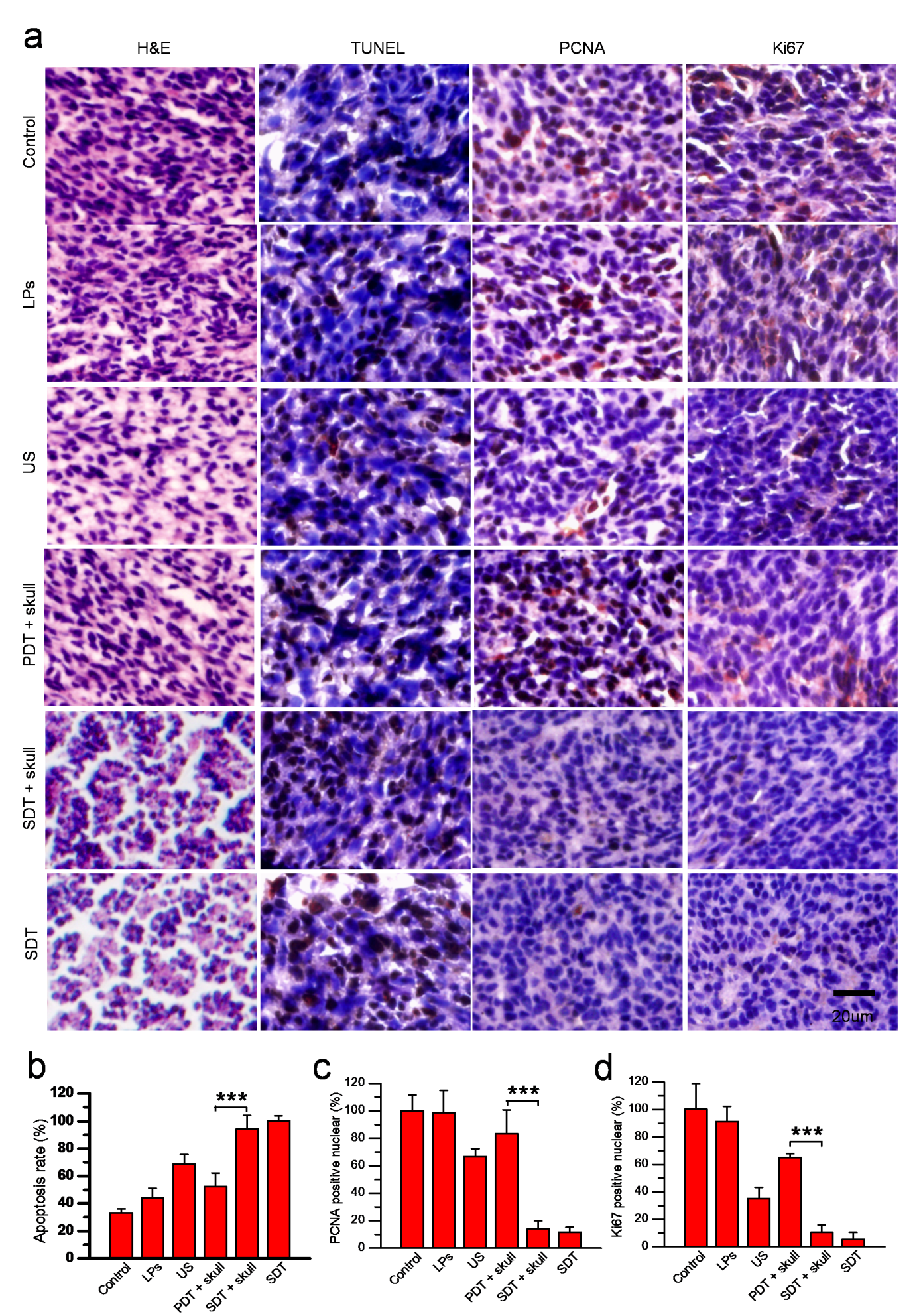


Figure S7. Histological examination of tumors after various treatments. (a) H&E staining, TUNEL staining, immunohistochemistry staining for PCNA and Ki67 of tumor sections collected from different treated groups of mice 24 h post treatment. The apoptotic cells were evidently identified by TUNEL assay (brown). Quantitative number of TUNEL-positive (b), PCNA-positive (c) and Ki67-positive cells (d) were determined by manually counting six random fields for each group. The data was shown as mean ± SD; (*) P < 0.05, (**) P < 0.01, (***) P < 0.001. ( Scale bar: 20 μm).
